# Conditioning attenuates kidney and heart injury in rats following transient suprarenal occlusion of the abdominal aorta

**DOI:** 10.1038/s41598-020-61268-9

**Published:** 2020-03-19

**Authors:** Dimitra M. Karageorgiadi, Diamantis I. Tsilimigras, Platonas Selemenakis, Vassiliki Vlachou, Anne-Lise de Lastic, Maria Rodi, Danai Chatziathanasiou, Konstantinos Savvatakis, Nikolaos Antoniou, Aikaterini C. Deli, Alexandros Papalampros, Konstantinos A. Filis, Athanasia Mouzaki, Anastasia Varvarigou, George Zografos, Vassilis G. Gorgoulis, Ioannis S. Pateras, Fragiska Sigala

**Affiliations:** 1 Molecular Carcinogenesis Group, Department of Histology and Embryology, School of Medicine, National and Kapodistrian University of Athens, Athens, Greece; 2 First Department of Propaedeutic Surgery, Hippocration General Hospital, School of Medicine, National and Kapodistrian University of Athens, Athens, Greece; 3 Department of Surgery, The Ohio State University Wexner Medical Center and James Cancer Hospital and Solove Research Institute, Columbus, OH USA; 4 Department of Environmental and Radiological Health Sciences, Colorado State University, Fort Collins, CO 80523 USA; 5 Laboratory of Immunohematology, Division of Hematology, Department of Internal Medicine, Medical School, University of Patras, Patras, Greece; 6 Department of Translational Oncology of Solid Tumors, German Consortium for Translational Cancer Research, German Cancer-research center (DKTK/DKFZ), Heidelberg, Germany; 7 First Department of Surgery, Laikon University Hospital, National and Kapodistrian University of Athens, Athens, Greece; 8 Department of Pediatrics, University of Patras Medical School, Patras, Greece; 9 Biomedical Research Foundation of the Academy of Athens, Athens, Greece; 10 Faculty of Biology, Medicine and Health Manchester Cancer Research Centre, Manchester Academic Health Sciences Centre, The University of Manchester, Manchester, UK

**Keywords:** Experimental models of disease, Translational research

## Abstract

Suprarenal aortic clamping during abdominal aortic aneurysm (AAA) repair results in ischemia-reperfusion injury (IRI) in local (i.e. kidney) and distant (i.e. heart) tissue. To investigate perioperative approaches that mitigate IRI-induced tissue damage, Wistar rats underwent suprarenal aortic clamping either alone or in combination with short cycles of ischemic conditioning before and/or after clamping. Serum analysis revealed significant reduction in key biochemical parameters reflecting decreased tissue damage at systemic level and improved renal function in conditioned groups compared to controls (p < 0.05), which was corroborated by histolopathological evaluation. Importantly, the levels of DNA damage, as reflected by the biomarkers 8-oxo-G, γH2AX and pATM were reduced in conditioned versus non-conditioned cases. In this setting, NADPH oxidase, a source of free radicals, decreased in the myocardium of conditioned cases. Of note, administration of 5-HD and 8-SPT blocking key protective signaling routes abrogated the salutary effect of conditioning. To further understand the non-targeted effect of IRI on the heart, it was noted that serum TGF-β1 levels decreased in conditioned groups, whereas this difference was eliminated after 5-HD and 8-SPT administration. Collectively, conditioning strategies reduced both renal and myocardial injury. Additionally, the present study highlights TGF-β1 as an attractive target for manipulation in this context.

## Introduction

Abdominal aortic aneurysm (AAA) is a potentially life-threatening entity, relatively common in routine clinical practice^1^. Despite the introduction of endovascular aortic aneurysm repair (EVAR) as well as the significant improvements in surgical techniques, grafts, and perioperative care during the last decades, AAA repair still carries a considerable risk of morbidity and mortality^2^. One of the main concerns in vascular surgery over the years has been the ischemia-reperfusion injury (IRI) generated by the suprarenal aortic clamping performed during AAA repair^2^. Although EVAR is nowadays considered the standard of care for most infrarenal AAA, open repair with aortic cross-clamping above the renal arteries is still the gold standard for juxtarenal aortic aneurysms^3^. The latter includes a temporal occlusion of the renal arteries with deprivation of blood supply to the kidneys for approximately 30–60 minutes during suprarenal aortic cross-clamping, which subsequently increases the risk of postoperative renal dysfunction^3^. Although the main targets of IRI seem to be the organs directly affected by the reduced blood supply (i.e. the kidneys, gastrointestinal tract and the lower limb muscles), recent evidence suggests the concurrent injury to distant organs including the myocardium^4^. Interestingly, AAA-repair associated mortality is mostly related to cardiac dysfunction^5^. An imbalance between metabolic supply and requirements during occlusion of arterial blood supply followed by sudden restoration of oxygen and nutrient supply leads to extended cellular damage. Indeed, there is considerable evidence suggesting that reactive oxygen and nitrogen species (ROS/RNS) play a pivotal role in the phenomenon of “reperfusion injury”^6^. Blood flow restoration to ischemic tissue leads to free radical accumulation, which subsequently leads to oxidative DNA damage^6^.

In 1984, Zager *et al*.^7^ demonstrated that a period of global ischemia rendered rat kidneys less susceptible to subsequent ischemic insults. Two years later, Murry *et al*.^8^ reported that brief cycles of ischemia-reperfusion applied prior to a prolonged episode of ischemia, collectively termed as Ischemic Preconditioning (IPre), have a protective effect on myocardial tissue. Since then, there have been multiple clinical and experimental studies on the subject of conditioning. The concept of IPre has evolved into “Ischemic Conditioning (IC)” during the past three decades, a term that encompasses a number of related endogenous protective strategies applied directly to the local, potentially affected organ including IPre and Ischemic Postconditioning (IPost) or to other remote organs [Remote Ischemic Preconditioning (RIPre), Remote Ischemic Postconditioning (RIPost)] that could be injured^9^. IPost was introduced by the group of Vinten-Johansen^10^ who applied three cycles of ischemia-reperfusion (30 seconds) after 60 minutes of coronary occlusion, demonstrating -for the first time- the cardioprotective effects of this procedure. Both IPre and IPost require direct interference with the blood vessels of the target tissues. On the other hand, the concept of remote conditioning encompasses an inter-organ protection, where ischemia in one vascular bed protects tissues supplied by other vascular beds^11^. In this frame, conditioning has been shown to display anti-oxidant properties, thus providing an explanation for the protective effect of this strategy on IRI^9^.

Although several studies have shown that remote conditioning effectively reduces the incidence of postoperative myocardial injury/infarction and renal impairment among patients undergoing open AAA repair, results in the literature are rather contradictory^12^. A recent systematic review and meta-analysis demonstrated the efficacy of IPost in preclinical studies; nevertheless, the significant heterogeneity of the studies included in this analysis limits the interpretation of these findings^13^. In addition, translating the experimental findings into clinical practice has been difficult to date. Hence, identifying effective strategies to reduce IRI is imperative. To this end, the objective of the current study was to investigate on both local (i.e. kidney) and remote (i.e. heart) organ tissues the following: (a) the effect of different perioperative conditioning strategies (IPre, IPost, and combined IPre and IPost) on tissue integrity by assessing key biochemical and histopathological parameters following suprarenal occusion of the aorta; (b) the impact of each strategy on the formation of modified Guanine at 8-O position (8-oxo-G) and the accumulation of phosphorylated histone H2AX at Serine 139 (also termed γH2AX) -two biomarkers reflecting oxidative DNA damage and the formation of Double Strand Breaks (DSBs), respectively- given that free radicals may cause potentially harmful lesions to DNA, and (c) the effect of blocking two key protective pathways involved in IRI on tissue integrity by administering the nonselective adenosine receptor antagonist 8-(p-Sulfophenyl) theophylline hydrate (8-SPT) and the mitochondrial ATP-sensitive (KATP) potassium channel blocker sodium 5-hydroxydecanoate (5-HD).

## Methods and Materials

### Animals

Adult male Wistar rats (body weight range 300–400 g) were purchased from the Hellenic Pasteur Research Institute and maintained in weather controlled chambers (Laboratory of Experimental Surgery and Surgical Research “N.S. Christeas”, Athens Medical School, First Department of Pathology, School of Medicine, National University of Athens) with free access to food and water. The protocol was approved by the Ethics Committee of the Athens Medical School and by the Veterinary Directorate of Attica Region in agreement with the Directive 2010/63/EU. The methods were carried out in accordance with the approved guidelines.

### Surgical procedure

Eighty Wistar male rats underwent the following surgical procedure; animals were anesthetized with a combination of ketamine (75 mg/kg) and xylazine (10 mg/kg) administered intraperitoneally. Animals were then placed in a supine position under a warm water flow temperature- regulated bed (EX-212; Euthanex Corp) in order to maintain core temperature at 37 °C ± 1 °C. The animals were intubated for mechanical oxygenation with a special respirator for small animals (MD Industries, Mobile, AL). The ventilator was adjusted to operate at a rate of 35 breaths/min. The animals underwent a laparotomy, and the abdominal aorta above the renal arteries was isolated. Temporary ligation silk was placed in the suprarenal aorta. All procedures were performed under an optical microscope at 10x magnification.

### Animal treatment

**Figure 1:**
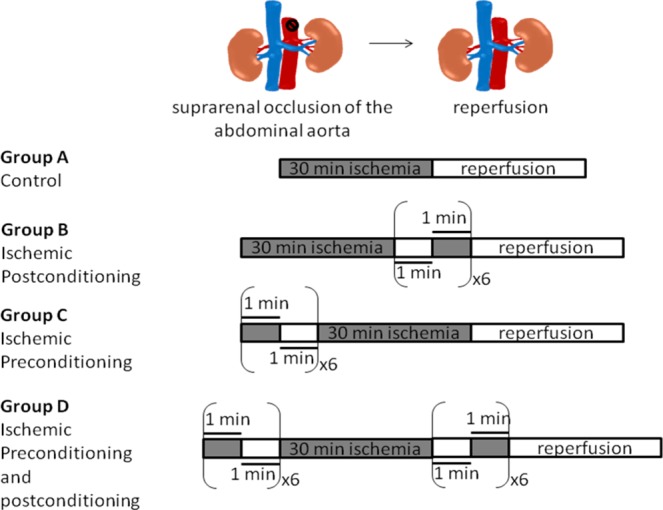
Schematic presentation of the experimental design and animal allocation into different groups. Group A (Control): occlusion of the suprarenal aorta for 30 min Group B (IPost): 30 min of suprarenal aortic occlusion prior to 6 cycles of 1 min ischemia – 1 min reperfusion. Group C (IPre): 6 cycles of 1 min ischemia – 1 min reperfusion prior to 30 min of occlusion. Group D (IPre + IPost): 6 cycles of 1 min ischemia – 1 min reperfusion and another 6 cycles of 1 min ischemia – 1 min reperfusion after prolonged ischemia. All animals in all groups were sacrificed at 6 h, 24 h, 48 h and 10d.

The animals were randomly divided into 4 groups and were subjected to 4 different types of treatment (Fig. 1): Group A: *Control group* (n = 20). The suprarenal aorta was occluded for 30 min. and the animals were sacrificed at 6 h, 24 h, 48 h and 10d. Group B: *Ischemic Postconditioning group - IPost* (n = 20). Thirty minutes (min.) of suprarenal aortic occlusion were followed by 6 cycles of 1 min ischemia – 1 min reperfusion and the animals were sacrificed at 6 h, 24 h, 48 h and 10d. Group C: *Ischemic preconditioning group - IPre* (n = 20). Six cycles of 1 min ischemia – 1 min. reperfusion was performed prior to 30 min. of suprarenal occlusion and the animals were sacrificed at 6 h, 24 h, 48 h and 10d. Group D: *Combination of ischemic preconditioning and postconditioning* group - *IPre* + *IPost* (n = 20). Prior to the 30 min of suprarenal aortic occlusion, the animals underwent 6 cycles of 1 min ischemia – 1 min reperfusion followed by 6 cycles of 1 min ischemia – 1 min reperfusion after the prolonged occlusion. Finally, the animals were sacrificed at 6 h, 24 h, 48 h and 10d by intravenous administration of sodium pentobarbital (300 mg, Dolethal, Vetoquinol UK Ltd, Buckingham, UK).

The nonselective adenosine receptor antagonist 8-SPT and the ATP-sensitive potassium channel blocker 5-HD were administered intraperitoneally separately in another series of animals exactly as above, 30 min prior to any procedure. The resulting experimental groups of the study will be henceforth identified as A, B, C, D; A_5-HD_, B_5-HD_, C_5-HD_, D_5-HD_ and A_8-SPT_, B_8-SPT_, C_8-SPT_, D_8-SPT_.

Blood was collected via cardiac puncture in vacutainer tubes (Becton Dickinson, BD), centrifuged at RT for 15 min at 3000 g, and serum stored in cryotubes at −80 °C. After sacrifice, tissues from different organs were quickly removed and divided in two parts: one part was fixed immediately in 10% neutral-buffered solution with 4% formaldehyde for 24 h before being embedded in paraffin, and the other part was immediately stored at −80 °C for further processing.

### Biochemical analysis

Serum levels of C-reactive protein (CRP), creatine phosphokinase-MB (CPK-MB), urea, creatinine (Cr), sodium (Na+) and potassium (K+), Lactic Acid Dehydrogenase (LDH), Glutamate-Pyruvate Transaminase (SGPT) were measured using a multianalyzer (Datebehring Dimension AR). Measurements of each group were performed in duplicate (SD <10%).

### Histopathological evaluation

The histopathological evaluation of rat kidney and heart was done by two experienced histopathologists. For the quantification of the tissue damage, a scoring system was employed based on previously published criteria^14–17^. Collective histological scoring ranged from 0 (within normal range) to 5 (severe organ injury).

### Immunohistochemistry (IHC)

The following antibodies were employed: anti-γH2AX (Ser139, 05–636, Millipore; 1:1000), anti-8-oxoguanine (8-oxo-G, MAB3560, Millipore; 1:750), anti-p65 (sc-372, Santa Cruz; 1:100) and anti-NOX4 (NB110-58851, Novus Biologicals, 1:200). For IHC the UltraVision^TM^ Quanto Detection System HRP DAB was employed (#TL-125-QHD, Thermo Scientific) according to the manufacturer’s instructions. Unmasking of the antigen retrieval was performed by heat-mediated antigen retrieval method in 10 mM citric acid (pH 6.0). The primary antibodies were incubated at 4 °C overnight. Hematoxylin was employed as counterstaining. A microscope (DM LB; Leica) equipped with a digital camera (DFC320; Leica) and N Plan objectives were used for picture acquisition. γH2AX, 8-oxo-G and p65 positivity was ascribed as previously described^18,19^. NOX4 evaluation was performed as previously described^20^. For γΗ2ΑΧ, 8-oxo-G, p65 and NOX4 previously characterized cases were employed as positive controls^18,19,21^. Slide examination was performed by three independent observers with minimal inter-observer variability.

### TUNEL assay

Tdt-mediated dUTP end labeling (TUNEL) assay has been performed as previously described^22^.

### Immunoblot analysis

Total protein extraction and Western blot analysis was performed as previously described^22^. *Histone extraction:* Subcellular fractions consisting of nuclear and membranous–cytoplasmic protein extracts were dissolved in histone Lysis Buffer (1 M Hepes pH 7.4, 0.5 M MgCl_2_, 1 M KCl, 100 mM DTT, 1 N HCl, protease and phosphatase inhibitors) and incubated for 1 h at 4 °C, followed by a 15 min. centrifugation at 13500 rpm. The supernatants were collected and the protein concentration was measured by the Bradford assay. Before gel electrophoresis, the pH of the samples was adjusted to 7 (neutral pH) by the addition of Tris buffer pH 10.

#### Antibodies

The following antibodies were used: anti-γH2AX (05-636, Ser139; Millipore; 1:1000), Histone H3 (C-16, Santa Cruz Biotechnology Inc; 1:1000), anti-NOX4 (NB110-58851, Novus Biologicals, 1:500), Phospho-ATM (10H11.E12, Ser1981, Santa Cruz Biotechnology Inc; 1:200–1:1000), β-actin (ΑC-15, ab6276; Abcam; 1:400). Histone H3 and β-actin served as loading controls.

### Senescence

Assessment of senescence was performed by a hybrid histo-/immuno chemical assay utilizing GL13, a lipophilic, biotin-linked Sudan Black-B (SBB) analogue (commercially available as SenTraGor®)^23^. Previously characterized cases served as positive controls^23^.

### TGF-β1 and MCP-1 Immunoassays

TGF-β1 and MCP-1 levels were measured by ELISA following the manufacturers’ instructions. For TGF-β1 the Quantikine^®^ ELISA #MB100B kit was used and for MCP-1 the Quantikine^®^ ELISA #MJE00 kit was used (R&D Systems).

### Statistical analysis

The results are presented as mean ± (SEM). Comparisons of numeric variables among groups were performed using ANOVA and Tukey’s multiple comparisons test. A p value of less than 0.05 was considered statistically significant.

## Results

### Improved profile in key biochemical markers in conditioned cases

**Figure 2:**
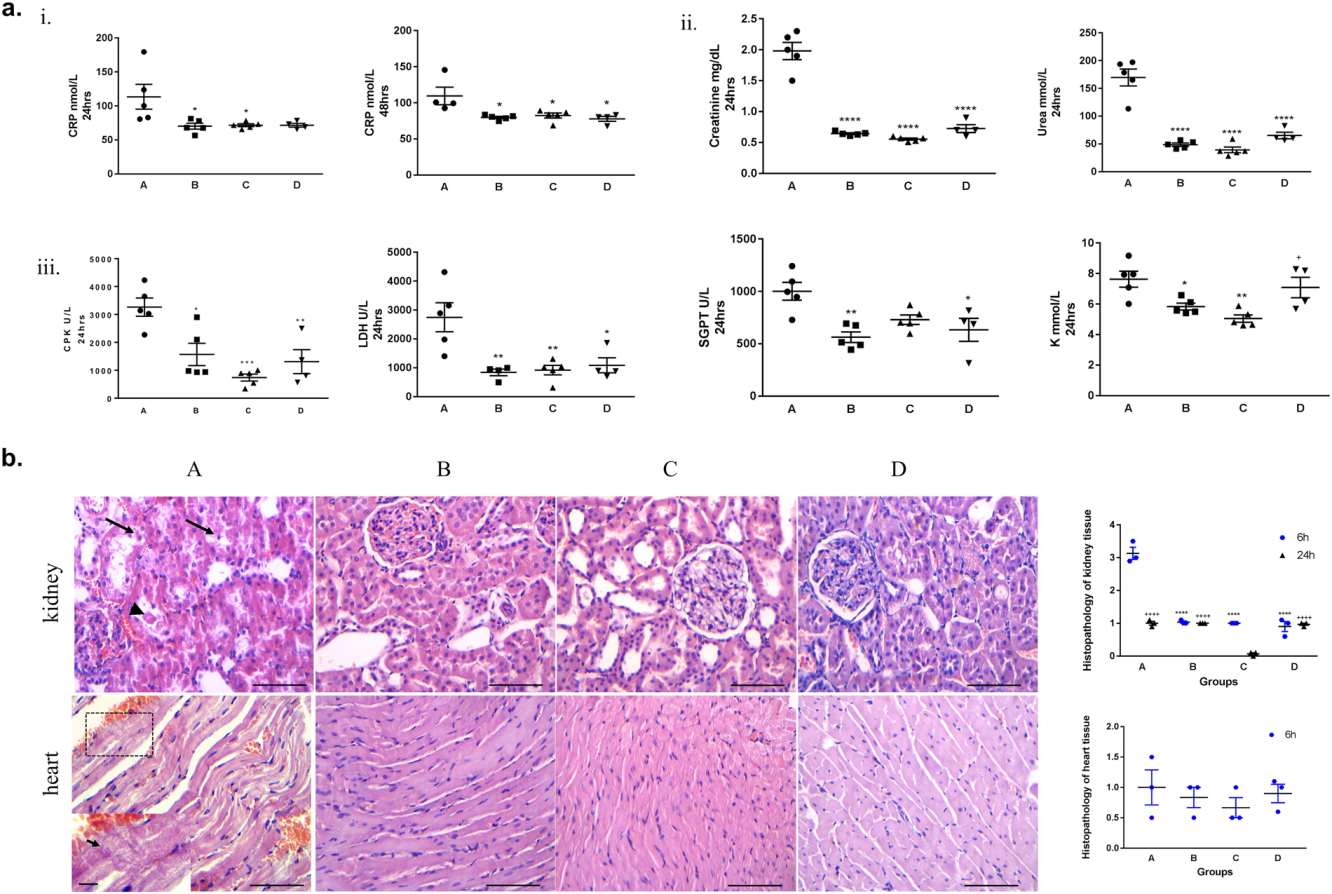
Conditioning exerts a protective effect on tissue integrity. (**a**) Improved profile in key biochemical markers in conditioned cases for 24 h and 48 h subgroup. i. Significant reduction in CRP indicating lower inflammation. ii. Significant decrease in urea and creatinine levels showing a protective effect on kidney function. iii. Significant reduction in CPK, LDH, SGPT and K^+^ levels reflecting reduced tissue damage. Data are expressed as mean ± SEM (n = 5), *p < 0.05, **p < 0.01, ***p < 0.001, ****p < 0.0001, ^+^p < 0.05 C vs D (one-way ANOVA with Turkey’s post hoc test). (**b**) Representative hematoxylin-eosin stained sections revealed reduced tissue damage in conditioned cases in the kidney and the heart in 6 h and 48 h subgroups. Both in kidney and heart the total scoring (ranging from 0–5) is improved in conditioned cases. Arrowhead depicts tubular crust and arrows demonstrate desquamate necrotic cells in the kidney (scale bar: 100 μm). Arrow within inset demonstrates contraction bands in the myocardium (scale bar: 100 μm; scale bar within inset 20 μm). Quantitative data are expressed as mean ± SEM (n = 3). For the kidney tissue, ****p < 0.0001 for 6 h; ^++++^p < 0.0001 for 24 h (two-way ANOVA with Turkey’s post hoc test).

To examine the effect of pre-, post- and combined pre- and post-conditioning on tissue integrity, we initially assessed the status of key biochemical parameters. Biochemical analysis in the serum of all groups sacrificed in 24 h and 48 h revealed a significant reduction in the acute phase protein C-Reactive Protein (CRP) in the conditioned groups (namely B, C, D; p < 0.05), indicating lower inflammation (Figs. 1 and 2ai). A significant decrease was also observed in urea and creatinine levels in the conditioned cases (p < 0.05) suggesting a protective effect of perioperative conditioning on kidney function (Fig. 2aii). Similarly, compared to non-conditioned group A, serum CPK, LDH, SGPT and K+ levels were lower in groups B, C and D reflecting reduced cellular lysis and tissue damage systematically in conditioned cases (Fig. 2aiii). Our results indicate that all types of ischemic conditioning lead to decreased inflammation and tissue injury.

### Conditioning lessens the histopathological lesions in kidney and heart

To further investigate the effect of each perioperative strategy in an organ-specific manner, we proceeded with histopathological examination of the kidney and heart. In non-conditioned cases, renal damage was prominent from the earliest time point (6 h), with loss of brush border, cytoplasmic vacuolization, necrotic cells and sloughing of the latter into the tubular lumen, and the formation of protein casts admixed with necrotic debris (Fig. 2b). Similarly, in the myocardium from non-conditioned rats, contraction band necrosis, a form or irreversible myocardial injury was evident from 6 h, accompanied by cellular vacuolization, focal interstitial edema and hemorrhagic infiltration (Fig. 2b). Histopathological examination of kidney and heart at additional time points further verified the protective effect of conditioning (Fig. 2b) although the differences in rats sacrificed 10d after ischemia were minimal among the various groups. Overall, our results show that ischemic conditioning decreases the histopathological lesions observed in non-conditioned cases both in kidney and heart corroborating the results from the biochemical analyses.

### Conditioning reduces the accumulation of DNA damage in kidney and heart

**Figure 3:**
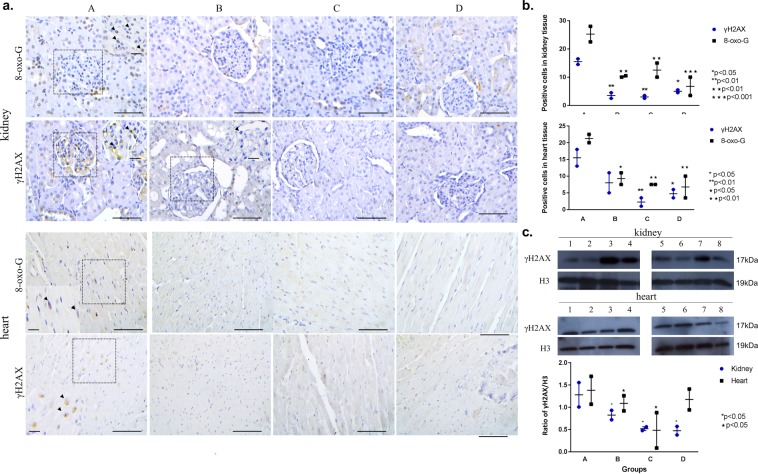
Decreased levels of DNA damage markers in conditioned cases. (**a**) Diminished 8-oxo-G and γH2AX immunostaining in representative conditioned cases in the kidney and the heart. Arrowheads depict positive γH2AX and 8-oxo-G immunostaining (scale bar: 100 μm; scale bars within insets: 25 μm). (**b**) Corresponding scatter plots depict the cumulative data of γH2AX and 8-oxo-G. For kidney tissue, quantitative data are expressed as mean ± SEM (n = 2), for 24 h subgroup. For heart tissue, quantitative data are expressed as mean ± SEM (n = 2), for 48 h subgroup (two-way ANOVA with Turkey’s post hoc test). (**c**) Western blot analysis for the DDR marker γH2AX in representative cases, demonstrating decreased γH2AX status in conditioned cases. H3 served as loading control. Quantification of western blot analysis of γH2AX/H3 ratios showed decreased levels in all conditioned groups which was more pronounced in group C. Data are expressed as mean ± SEM (n = 2) (two-way ANOVA with Turkey’s post hoc test). Kidney samples: 1:B6, 2:B9, 3:A8, 4:A10, 5:C13, 6:C14, 7:D6, 8:D9; heart samples 1:A11, 2:B13, 3:B15, 4:A15, 5:D9, 6:D10, 7:C12, 8:C13. A_6–15_, B_6–15_, C_6–15_, D_6–15_ samples represent 24 h and 48 h subgroups.

A critical factor in reperfusion injury following blood flow restoration is the excess production of free radicals^6^. To assess oxidative DNA damage, we examined the status of 8-oxo-G, a major product of oxidative damage reflecting cellular oxidative stress^24^. The levels of 8-oxo-G were significantly reduced in all conditioned versus non-conditioned cases from 24 h and 48 h subgroups (Fig. 3ai, ii). Within this frame, assessment of NADPH oxidase (NOX4), an important enzymatic source of ROS in cardiomyocytes^25^, demonstrated elevated NOX4 levels in group A versus conditioned cases (Supplementary Fig. 1ai, ii). The latter finding comes in line with the increased levels of 8-oxo-G in unconditioned Group A.

Given that free radicals can in turn cause strand breaks in DNA^26^, we examined the status of γH2AX, a prototypic biomarker that is accumulated upon DSBs^27^, along with the levels of phosphorylated ATM kinase at Serine 1981 (designated as pATM), a protein kinase that is activated upon DSB formation^28^ (Fig. 3ai, ii). Conditioning was associated with reduced levels of γH2AX both in kidney and heart in 24 h and 48 h subgroups, further supporting the protective effect of this strategy. Western blot analysis for γH2AX and pATM supported our immunohistochemical findings, verifying the protective effect of perioperative conditioning on DNA integrity (Fig. 3aiii and Supplementary Fig. 1b). In addition, we observed reduced apoptosis in the conditioned cases in 48 h subgroup that comes in line: a) with the decreased activation of DNA damage response and repair machinery (DDR/R) reflected by the status of γH2AX and pATM (further analyzed in the Discussion) and b) with the reduced histopathological lesions in these cases (Supplementary Fig. 1c). Additionally, given that activation of DDR/R may also trigger senescence, we utilized a novel hybrid histo-/immuno chemical assay employing GL13, a lipophilic biotin-linked Sudan Black-B (SBB) analogue (commercially available as SetTraGor®), which reacts with lipofuscin, a non-degradable material that accumulates in senescent cells^23^. GL13 staining did not reveal significant differences in our setting (Supplementary Fig. 1d). Collectively, our results demonstrate the protective effect of ischemic conditioning on DNA integrity by assessing the accumulation of 8-oxo-G as well as two sensitive markers of DSBs both in the kidney, a directly affected organ by abdominal aortic occlusion, and in the heart, distant to the occlusion.

### Conditioning decreases the levels of serum TGF-β1

The non-targeted effect of IRI on the heart is indicative of the existence of mediators released from the directly affected site and induce DNA damage at distant sites. We focused our analysis on the effect of conditioning on TGF-β1 serum levels for the following reasons: a) TGF-β1 mRNA levels are elevated following ischemia injury in rat kidney^29^, b) TGF-β1 has a central role in myocardial homeostasis after ischemia and reperfusion^30^, c) TGF-β1 induces ROS production mainly through NOX4, and ROS in turn favors TGF-β1 activity forming a vicious cycle^31^ and d) TGF-β1 can induce DSBs in bystander cells in a ROS-dependent manner^32^. As expected, group B, C and to a greater degree group D cases exhibited lower levels of serum TGF-β1 (Supplementary Fig. 2a), which is in accordance with the decreased status of 8-oxo-G, γH2AX, pATM and NOX4 in kidney and heart of conditioned cases. The diminished nuclear immunostaining of p65, a downstream target of TGF-β1^31,33^, in the myocardium of representative conditioned cases further comes in line with TGF-β1 levels in these cases (Supplementary Fig. 2b). Additionally, prompted by previous findings linking Monocyte Chemoattractant Protein 1 (MCP-1) [alternatively knows as chemokine (C-C) ligand 2 (CCL2)] with DNA damage in out-of-field tissues following irradiation^34^, we examined the status of MCP-1/CCL2 in our setting but found no differences among the conditioned and unconditioned groups (Supplementary Fig. 2c**)**. Collectively, our findings indicate that TGF-β1 can play a role in myocardial IRI after abdominal aortic occlusion.

### Treatment with 5-HD or 8-SPT abolishes the protective effect of conditioning

**Figure 4:**
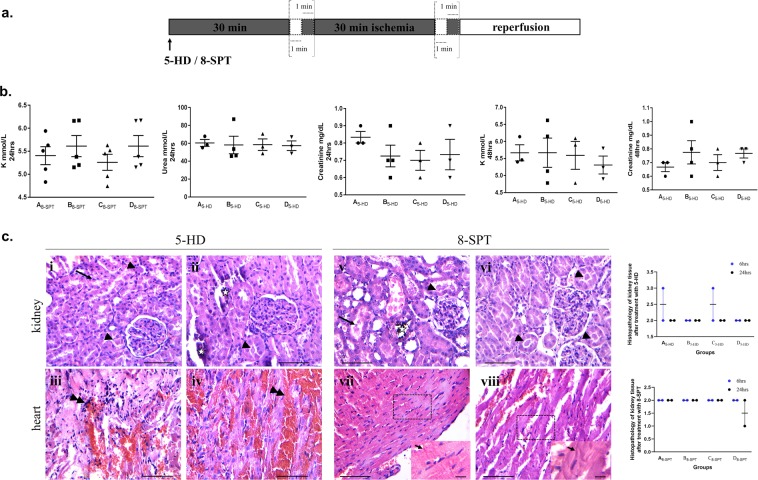
Treatment with 5-HD or 8-SPT abolishes the protective effect of conditioning. (**a**) Schematic presentation of the experimental design. 5-HD or 8-SPT was administrated prior to suprarenal occlusion of the abdominal artery and 6 cycles of 1 min ischemia – 1 min reperfusion. (**b**) No significant differences in the levels of key biochemical parameters are noted including K^+^, creatinine, urea between conditioned and un-conditioned cases upon 5-HD or 8-SPT treatment (one-way ANOVA with Turkey’s post hoc test). (**c**) Representative hematoxylin-eosin stained sections demonstrate significant tissue damage in 5-HD or 8-SPT treated conditioned cases. Tubular crusts (arrowheads) along with desquamate necrotic cells (arrows) and calcification (asterisks) in the cortex of the kidney from representative post-conditioning (i, v), pre-conditioning (vi) and combined pre- and post-conditioning (ii) cases (scale bars: 100 μm). Hemorrhagic along with inflammatory cell infiltrate (double arrowheads) and contraction bands (arrows within insets) in the myocardium from representative post-conditioning (iii, vii), pre-conditioning (viii) and combined pre- and post-conditioning (iv) cases (scale bars: 100 μm; scale bar within insets: 20 μm). Histologic score ranges from 0 to 5. Quantitative data did not show any statistical significance (two-way ANOVA with Turkey’s post hoc test).

Earlier studies have shown that administering the KATP channel blocker 5-HD or the non-selective adenosine inhibitor 8-SPT, eliminates the protective effect of IPre and IPost^35,36^. To decipher the role of mitochondrial KATP channel activity and adenosine signaling in this context, we administered 5-HD or 8-SPT before the application of suprarenal abdominal artery occlusion and reperfusion (Fig. 4a). Treatment with 5-HD and 8-SPT resulted in increased levels of urea, creatinine as well as K +in conditioned cases (Fig. 4b). Within this frame, histopathological evaluation revealed significant tissue damage in kidney and heart in 5-HD and 8-SPT treated rats irrespective of the perioperative strategy followed (Fig. 4c). To this end, there was no statistically significant difference among the different groups (A_8-SPT_, B_8-SPT_, C_8-SPT_, D_8-SPT_) after treatment with 8-SPT with respect to 8-oxo-G and γH2AX status (Supplementary Fig. 3) in 48 h subgroup, which comes in line with the histopathological evaluation. Similar findings were observed in cases with 5-HD treatment (data not shown). Therefore, administration of 5-HD or 8-SPT abrogates the protective effect of conditioning both in kidney and heart. The examination of serum TGF-β1 levels showed similar levels in the different time points among unconditioned (A_5-HD_, and A_8-SPT_) and conditioned (B_5-HD_, C_5-HD_, D_5-HD,_ B_8-SPT_, C_8-SPT_, and D_8-SPT_) groups (Supplementary Fig. 4) further supporting a potential role of TGF-β1 in IRI context.

## Discussion

ΙRΙ is associated with increased morbidity, incidence of myocardial infarction, stroke and acute renal injury^2^. Although, renal and heart IRI has been extensively studied, the underlying molecular events are still unclear. The present work contributes to our understanding of the impact of various conditioning strategies on directly (i.e. kidney) and remotely (i.e. heart) affected organs. Of note, the current study revealed that IPre, IPost and combined (IPre + IPost) conditioning resulted in improved biochemical profile as reflected by improved kidney function and reduced tissue destruction at a systemic level. Of note, consistent to reported elevated CRP levels in acute kidney injury^37^, CRP levels in all conditioned groups were decreased compared to the control group (i.e. group A) indicating an attenuated inflammatory response in the former cases. The histopathological analysis in kidney and heart further corroborated our findings demonstrating that all different conditioning procedures conveyed a protective effect. Our findings come in line with a review and meta-analysis by Wever *et al*. in 2012^38^ on 58 experimental animal studies showing that IPre reduced the levels of creatinine, blood urea nitrogen and histological damage compared to untreated animals which was more pronounced in the “second window of protection” (SWOP). SWOP refers to a delayed form of protection appearing after 24 h to 48 h upon IPre^39^.

Previous reports have demonstrated the accumulation of the oxidative DNA damage marker 8-oxo-G in kidney tissues following ischemia reperfusion^40^. Herein, we showed -for the first time- that different conditioning strategies result in reduced 8-oxo-G levels both in kidney and heart. Within this context, we further provided evidence that NOX4 is down-regulated in the myocardium of conditioned cases, which is in line with the role of NOX4 as a significant source of free radicals during myocardial ischemia-reperfusion^41^.

To this point, we showed that conditioning reduces oxidative DNA modifications. Taking into consideration a previous study that demonstrated an elevation of γH2AX and pATM in kidney tissue during renal IRI^42^, one would expect a decreased DDR/R activation following IC. Congruent to our hypothesis, γH2AX and pATM levels were decreased across all conditioned groups in comparison to group A both in kidney and heart. γH2AX and pATM are sensitive and robust biomarkers accumulated upon DSBs, indicating for the first time that IC exerts a DNA-protective effect. Previous evidence suggests that inhibition of Poly(ADP ribose) synthase (PARS) [also known as Poly(ADP ribose) polymerase, PARP], a critical component of DDR/R^28^, protects against myocardial IRI^43^. As such, our data provide additional evidence for the significance of DDR/R in IRI and IC. In addition, based on the findings of this study, apoptosis was also reduced in conditioned cases further highlighting the protective effect of this strategy which is in line with previous studies^44^.

Another interesting finding of our study is that TGF-β1 was reduced in all IC groups compared with the IRI group (i.e. group A). Notably, this difference was more pronounced in group D where a combined conditioning (IPre + IPost) was applied. TGF-β1 is a pleiotropic cytokine synthesized by a variety of cells and is implicated in a number of cellular functions^45^. Accumulating evidence has demonstrated that TGF-β1 may be up-regulated following acute ischemia having a salutary effect in kidney and heart tissue after IRI^29,30,46–48^. These observations emphasize the beneficial role of TGF-β1. This may at first glance seem counterintuitive with our findings; yet, although up-regulation of TGF-β1 may have a protective effect to some extent, it might exert an adverse effect on cellular homeostasis -when rising above a certain threshold- by inducing redox imbalance. This can be justified by the fact that TGF-β1 induces ROS production in certain contexts. In particular, TGF-β1 signaling has been implicated in the production of mitochondrial ROS through NOX4 induction^31,49^. Hence, the observed reduced TGF-β1 levels after conditioning in our setting, which was pronounced in the combined pre- and post-conditioning, may favor the protective effect of this cytokine on tissue integrity. In a similar manner, free radicals have a bivalent nature in IC; excess levels of ROS cause tissue damage, whereas small amounts protect cellular functions^50^. To this end, the IRI biology is highly reminiscent of the complexities in radiation-induced bystander/distant effect biology^34^. Within this frame, a systemic response has been depicted in out-of-field tissues upon irradiation verified by γH2AX foci suggesting DNA damage. A hint for the underlying mechanism comes from the elevated circulating levels of certain cytokines including TGF-β1, which may play a role as mediators of non-targeted effects by promoting oxidative DNA damage at distant sites^34^.

Previous studies have demonstrated that mitochondrial KATP channel activity and adenosine signaling exert a protective effect during IRI^9,35,36,51^. Induction of these molecular routes exert an immunomodulatory and anti-oxidant effect in different models of IRI^52–55^. Therefore, to gain a further insight in the pathogenesis of tissue damage in our context, we separately abrogated these two signaling pathways. Administration of the nonselective adenosine receptor antagonist 8-SPT or the mitochondrial KATP channel blocker 5-HD abrogated the protective effect of IC. Destruction of renal architecture accompanied by impairment of kidney function along with myocardium damage was clearly evident irrespective of the conditioning strategy. In view of the fact that NOX4 and mitochondrial KATP channel form a feed-forward loop in cardiac biology^56^, our findings pinpoint the involvement of this axis in the pathophysiological processes of IRI. Notably the serum levels of TGF-β1 were similar in all groups irrespective of the perioperative approach after treatment with 8-SPT or 5-HD, suggesting the involvement of both signaling pathways in the release of TGF-β1. Previous studies have linked the adenosine signaling with TGF-β1 status in different contexts^57,58^; however, further research is needed in order to clarify the relative contribution of mitochondrial KATP channel activity and the different types of adenosine-adenosine receptor in the induction of TGF-β1 in the particular setting.

Collectively, these data demonstrate the adverse effect of suprarenal aortic occlusion at organismal, cellular and molecular level and highlight the beneficial role of different conditioning procedures on both kidney and heart, a directly and remotely affected organ, respectively. In addition, this work indicates that TGF-β1 can be a potential mediator of the remote effects in this context. In light of the recent evidence demonstrating a beneficial systemic immune response and higher overall survival of metastatic breast cancer patients receiving TGFβ blockade during radiotherapy^59^, TGFβ1 becomes an attractive target with promising prognostic and therapeutic applications in IRI context. Hence, knowledge of IRI biology could be applied not only to understand the etiology of tissue damage during IRI but also to overcome current challenges in translating the experimental data into the clinical setting. To this end, the promising results yielded from clinical trials that examine transient perioperative episodes of ischemia as well as the evidence from preclinical studies suggest a protective role of the conditioning strategies^60^ and highlight the opportunity for clinical application in the setting of AAA repair.

## Supplementary information

Supplementary Figures.
