# Supplementary material for: Conditioning attenuates kidney and heart injury in rats following transient suprarenal occlusion of the abdominal aorta
Source: Sci Rep. 2020 Mar 19;10:5040. doi: 10.1038/s41598-020-61268-9 (PMC7081351; doi:10.1038/s41598-020-61268-9)
Supplement: Supplementary file 1 — Supplementary Figures. [file 41598_2020_61268_MOESM1_ESM.pdf]

Conditioning attenuates kidney and heart injury in rats following transient suprarenal occlusion of the abdominal aorta

Dimitra M. Karageorgiadi, Diamantis I. Tsilimigras, Platonas Selemenakis, Vassiliki Vlachou, Anne-Lise de Lastic, Maria Rodi, Danai Chatziathanasiou, Konstantinos Savvatakis, Nikolaos Antoniou, Aikaterini C. Deli, Alexandros Papalampros, Konstantinos A. Filis, Athanasia Mouzaki, Anastasia Varvarigou, George Zografos, Vassilis G. Gorgoulis, Ioannis S. Pateras, Fragiska Sigala

Supplemental Figures

Supplementary Figure 1

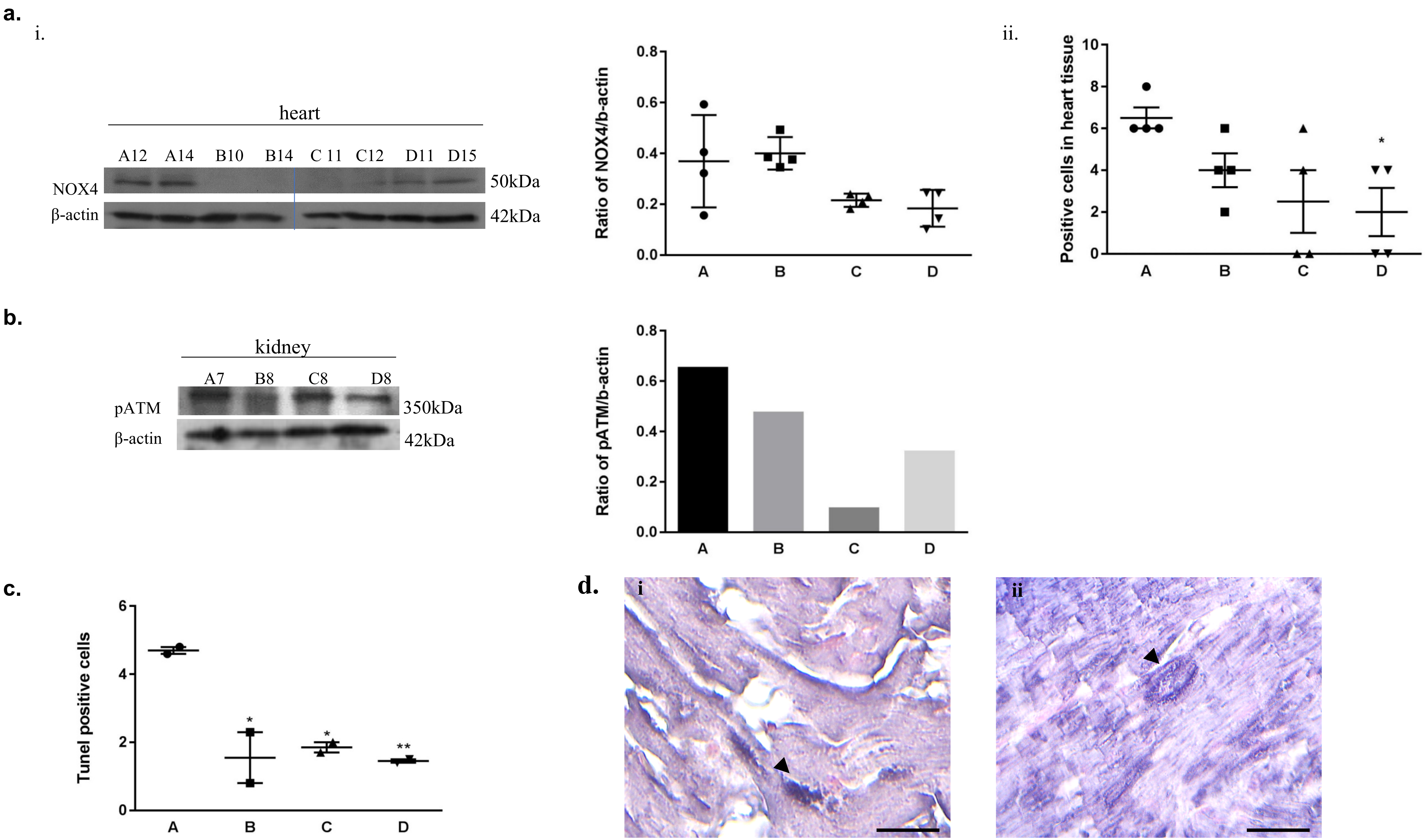

Supplementary Figure 1. (a) i. Western blot showing decreased NOX4 in the myocardium of conditioning cases in 48h subgroup. B-actin served as loading control. Quantification of western blot analysis of NOX4/β-actin ratios showed decreased levels in conditioned groups (one-way ANOVA with Turkey's post hoc test) Aii. Scatter plot demonstrating decreased NOX4 levels employing immunohistochemistry in the myocardium of conditioned cases in 48h subgroup. Data are expressed as mean ± SEM. *p<0.05 (one-way ANOVA with Turkey's post hoc test). (b). Western blot analysis for the DDR marker pATM in representative cases, demonstrating decreased pATM status in conditioning cases. B-actin served as loading control. Quantification of western blot analysis of pATM / β-actin ratios showed decreased levels in all conditioned groups which was more pronounced in group C in 24h subgroup. A6-15, B6-15, C6-15, D6-15 samples represent 24h and 48h subgroups. (c). Assessment of apoptosis in kidney employing TUNEL, demonstrated decreased number of apoptotic cells in conditioning cases in 48h subgroup. Data are expressed as mean ± SEM. *p<0.05, **p<0.01 (one-way ANOVA with Turkey's post hoc test) (d). Examination of senescence utilizing GL13, a lipophilic, biotin-linked SBB analogue (commercially available as SenTraGor®) in 48h subgroup. Arrows demonstrate GL13 positive cells from a representative case from Group A (i) and Group D (ii). Scale bar: 25μm.

Supplementary Figure 2

a.

i.

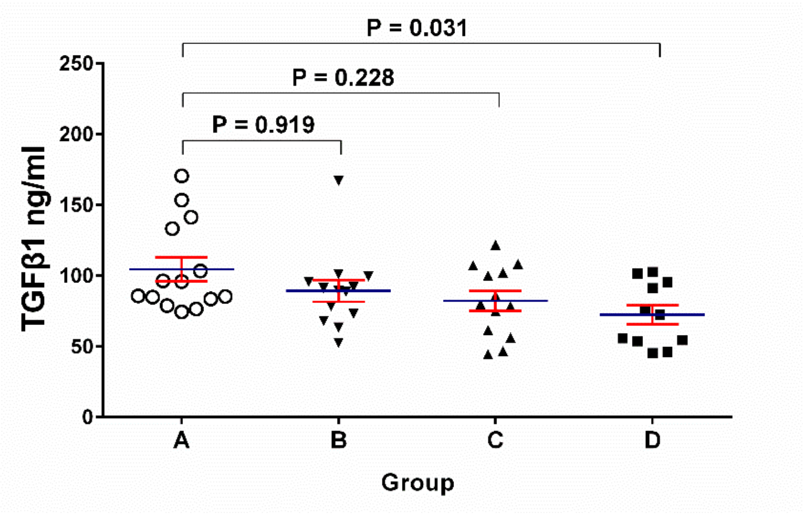

c.

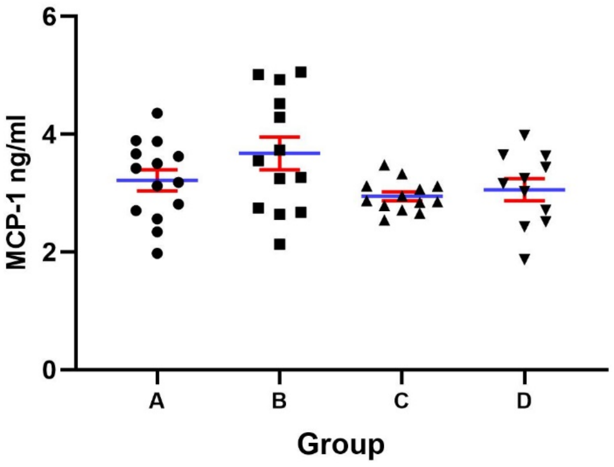

b.

i.

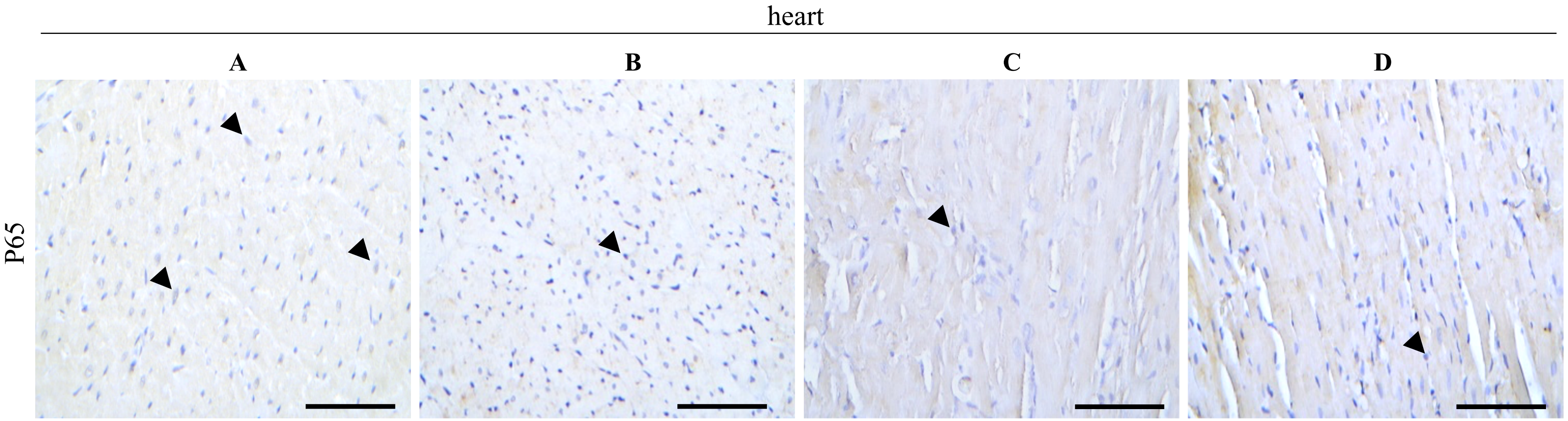

ii.

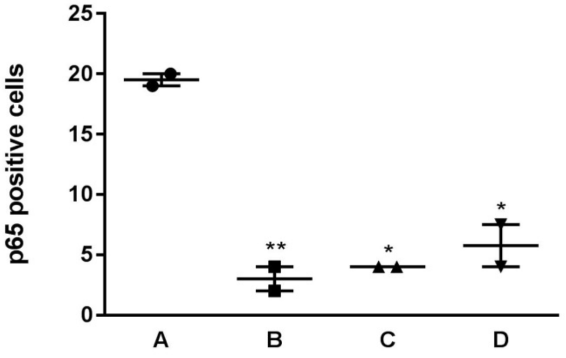

Supplementary Figure 2. **(**a). Decreased levels of TGFβ1 levels in the serum of pre-, post- and combined pre- and post-conditioning versus non-conditioning cases which is more prominent in Group D ($p=0.031$). Data are expressed as mean ± SEM (one-way ANOVA with Turkey's post hoc test). (b). Decreased nuclear staining for p65 in the myocardium of conditioning cases (scale bars: 100μm). Corresponding scatter plots depict the average status of p65 nuclear immunostaining. Data are expressed as mean ± SEM ($n=2$), *$p<0.05$, **$p<001$ A vs B, C, D for 24h (one-way ANOVA with Turkey's post hoc test). (c). No significant differences in CCL2/MCP-1 serum levels between Groups A, B, C, and D. Data are expressed as mean ± SEM (one-way ANOVA with Turkey's post hoc test).

Supplementary Figure 3

i.

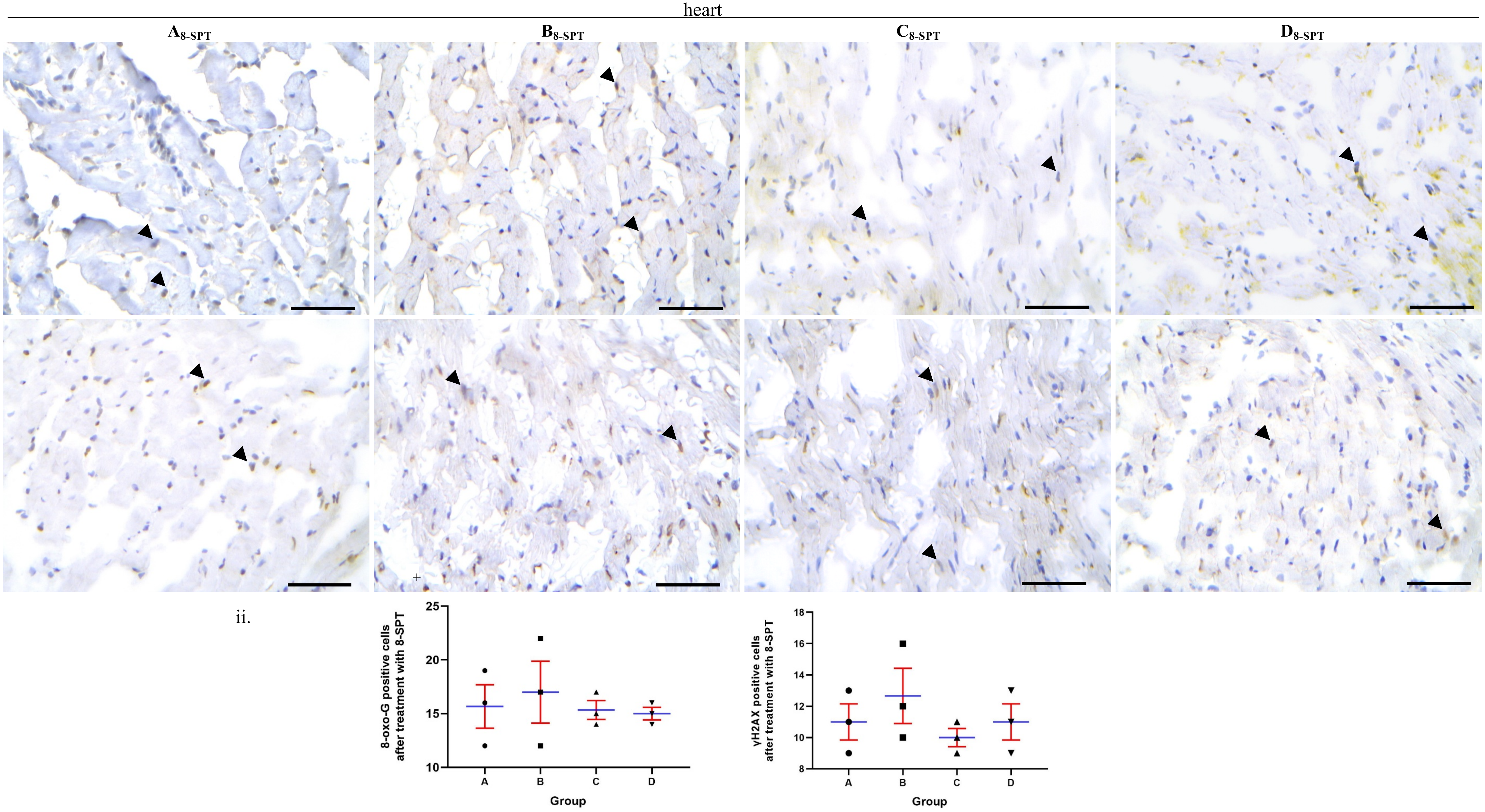

Supplementary Figure 3. No statistically significant difference between unconditioned (A8-SPT) versus conditioned (B8-SPT, C8-SPT, D8-SPT) cases after treatment with 8-SPT in the status of 8-oxo-G and γH2AX immunostaining in the myocardium. i. γH2AX and 8-oxo-G immunostaining in representative cases (scale bar: 100μm). Arrowheads demonstrate immunopositivity for the corresponding markers. ii. Scatter plots depict the cumulative data of γH2AX and 8-oxo-G. For heart tissue, quantitative data are expressed as mean ± SEM (n=2), for 48h subgroup (one-way ANOVA with Turkey's post hoc test).

Supplementary Figure 4

i.

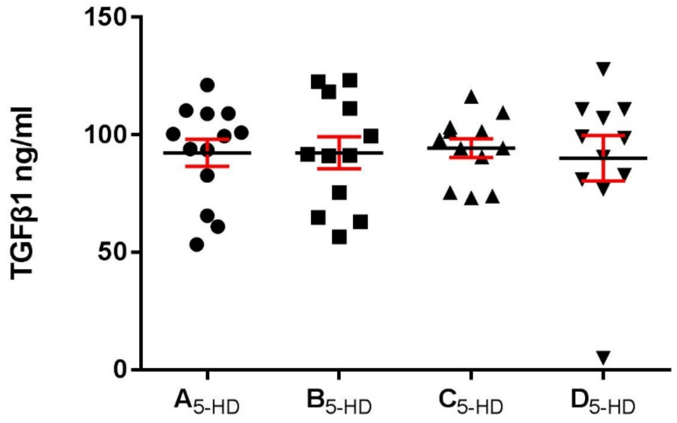

ii.

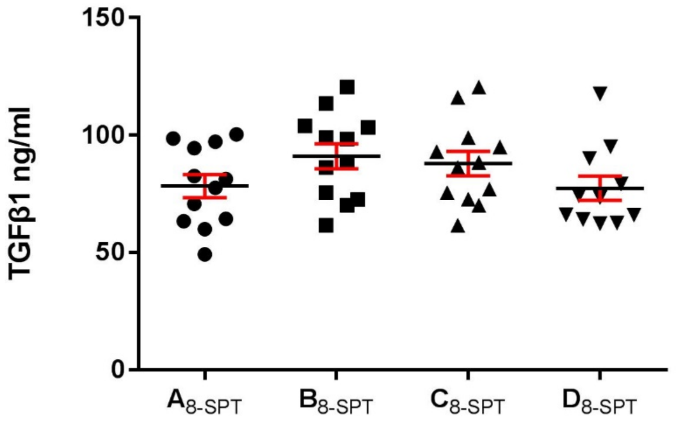

Supplementary Figure 4. Non-significant differences of TGFβ levels in the serum of pre-, post- and combined pre- and post-conditioning versus non-conditioning cases upon (i) 5-HD and (ii) 8-SPT treatment (one-way ANOVA with Turkey’s post hoc test).
